# What Is Apoplexy?

**Published:** 1870-09

**Authors:** 


### WHAT IS APOPLEXY?

From the suddenness of the attack and the apparent causelessness of it, the Greeks connected it in their own minds with the idea of a stroke of lightning as coming from the Almighty hand; it literally means, " A stroke from above." As instantaneous as the hurling of a thunderbolt in a clear sky, there comes a loss of sense, and feeling, and thought, and motion; the heart beats, the lungs play, but that is all, and soon they cease forever. The Romans considered the person to be —

" THUNDER STRUCK,"

or " planet struck; " as if it were of an unearthly origin.

The essential nature of apoplexy is an unnatural amount of blood in the brain; whatever sends too much blood to the brain, may cause apoplexy; whatever keeps the blood coming from the brain, dams it up, may cause apoplexy; that is the kind of apoplexy which seems to come without any apparent adequate cause. Tying a cord tightly around the neck, or holding the head downwards too long, can bring on an attack of apoplexy, by damming up the blood in the brain, and keeping it from returning to the body.

A sudden mental emotion can send too much blood to the brain; or too great mental excitement does the same thing. It is the essential nature of all wines and spirits to send an increased amount of blood to the brain; hence alcohol is said to stimulate the brain. The first effect of taking a glass of wine or stronger form of alcohol, is to send the blood there faster than common, hence it quickens the circulation; that gives the red face; it increases the activity of the brain, and it works faster, and so does the tongue. But as the blood goes to the brain faster than common, it returns faster, and no special permanent harm results. But suppose a man keeps on drinking, the blood is sent to the brain so much faster, in such larger quantities, that in order to make room for it, the arteries have to enlarge themselves; they increase in size, and in doing so, press against the more yielding, flaccid veins, which carry the blood out of the brain, and thus diminish their size, their bores; the result being, that the blood is not only carried to the arteries of the brain faster than is natural or healthful, but it is prevented from leaving it as fast as usual; hence, a double set of causes of death are set in operation. Hence, a man may drink enough brandy or other spirits in a few hours or even minutes to bring on a fatal attack of apoplexy; this is literally being dead drunk.

